# The mosaic of AII amacrine cell bodies in rat retina is indistinguishable from a random distribution

**DOI:** 10.1017/S0952523822000025

**Published:** 2022-05-10

**Authors:** Jian Hao Liu, David Olukoya Peter, Maren Sofie Faldalen Guttormsen, Md Kaykobad Hossain, Yola Gerking, Margaret Lin Veruki, Espen Hartveit

**Affiliations:** Department of Biomedicine, University of Bergen, Bergen, Norway

**Keywords:** amacrine cells, retina, random distribution, nearest-neighbor distance, Dirichlet domain

## Abstract

The vertebrate retina contains a large number of different types of neurons that can be distinguished by their morphological properties. Assuming that no location should be without a contribution from the circuitry and function linked to a specific type of neuron, it is expected that the dendritic trees of neurons belonging to a type will cover the retina in a regular manner. Thus, for most types of neurons, the contribution to visual processing is thought to be independent of the exact location of individual neurons across the retina. Here, we have investigated the distribution of AII amacrine cells in rat retina. The AII is a multifunctional amacrine cell found in mammals and involved in synaptic microcircuits that contribute to visual processing under both scotopic and photopic conditions. Previous investigations have suggested that AIIs are regularly distributed, with a nearest-neighbor distance regularity index of ~4. It has been argued, however, that this presumed regularity results from treating somas as points, without taking into account their actual spatial extent which constrains the location of other cells of the same type. When we simulated random distributions of cell bodies with size and density similar to real AIIs, we confirmed that the simulated distributions could not be distinguished from the distributions observed experimentally for AIIs in different regions and eccentricities of the retina. The developmental mechanisms that generate the observed distributions of AIIs remain to be investigated.

## Introduction

The narrow-field AII amacrine cell seems to be present in all mammalian retinas (for reviews, see Demb & Singer, 2012; Diamond, 2017) and of the >60 types of amacrine cells (Yan et al., 2020) is the most numerous, constituting 10–11% of the amacrine cell population (Strettoi & Masland, 1996; Pérez de Sevilla Müller et al., 2017). The cell bodies of AII amacrines are located in the first (proximal) tier of the inner nuclear layer and are typically slightly displaced into the neighboring inner plexiform layer. A small number of processes emanate directly from the cell body, one of which, the apical dendrite, is much thicker than the others and descends vertically into the inner plexiform layer. Here, the apical dendrite gives rise to two different types of dendritic processes, arboreal and lobular dendrites, which branch in the proximal and distal strata of the inner plexiform layer, respectively (Kolb & Famiglietti, 1974; Famiglietti & Kolb, 1975). Arboreal dendrites receive chemical synaptic input from the axon terminals of rod bipolar cells and make electrical synapses (*via* gap junctions) with arboreal dendrites of neighboring AII amacrine cells and axon terminals of ON-cone bipolar cells (Strettoi et al., 1992; Veruki & Hartveit, 2002*a*, *b*). Lobular dendrites make glycinergic synapses onto axon terminals of OFF-cone bipolar cells and dendrites of OFF-ganglion cells and are themselves postsynaptic to glutamatergic axon terminals of some OFF-cone bipolar cells (Strettoi et al., 1992; Veruki et al., 2003; Graydon et al., 2018; Hartveit et al., 2019). In addition to these microcircuits, the AII also takes part in synaptic relationships with a large number of different types of neurons in the retina (Marc et al., 2014), making the AII a multifunctional amacrine cell that plays a crucial role in visual signal processing under both scotopic and photopic conditions (Demb & Singer, 2012).

For most types of retinal neurons, the specific role and contribution to visual processing is independent of the location in the retina and thereby in the visual field. Although there are some well-documented exceptions to this general principle, for example, the almost exclusive localization of M- and S-cones to the dorsal and ventral halves, respectively, in mouse retina (Szél et al., 1992), and the almost exclusive localization of somatostatin-containing amacrine cell bodies to the inferior part of rabbit retina (Sagar, 1987), these pertain to a global, as opposed to a local, level of organization. At a more local level, it would seem that for any given type of neuron, no retinal location should be without a contribution of the retinal circuitry and processing capacity offered by that cell type (for review, see Cook, 2003; Wässle, 2004). Apart from extrasynaptic diffusion of neurotransmitter, the functional contribution to visual processing of an individual neuron will be linked to the area or volume over which the neuron distributes its processes. For the majority of (postreceptoral) retinal neurons, this corresponds to the distribution of dendritic processes. This means that the coverage factor (i.e., the number of dendritic fields covered by a single point on the retinal surface, calculated as the product of density and dendritic field size), is expected to equal or exceed 1 for all types of retinal neurons (for review, see Reese, 2008*b*). With the reasonable assumption that the contribution of each cell type to the local circuitry will be uniform across the retina, or at the very least be relatively uniform within, and vary gradually between, local regions, there is the expectation of orderly distribution and regular spacing of neurons of a given type across the retina. For a number of different types of retinal neurons, such local order has indeed been observed as orderly patterned arrays of uniform distribution, generally termed retinal mosaics (Reese, 2008*b*). The regularity within such mosaics is typically analyzed and expressed by simple spatial statistics, for example, the distribution of nearest-neighbor distances (Wässle & Riemann, 1978; Peichl & Wässle, 1981; Wässle et al., 1981; for review, see Cook, 1996; Reese, 2008*b*). For AII amacrine cells, presumed regular spacing has been observed in a number of species, for example, cat (Vaney, 1985), rat (Wässle et al., 1993), rabbit (Mills & Massey, 1991; Vaney et al., 1991; Casini et al., 1995), monkey/macaque (Wässle et al., 1995), bat (Jeon et al., 2007), and mouse (Pérez de Sevilla Müller et al., 2017). For the mouse retina, however, it was recently reported that AII amacrine cells display a soma distribution that cannot be distinguished from a simulated random distribution that is matched for density and constrained by soma size (Keeley & Reese, 2018). Although species differences cannot be excluded (see e.g., Martin et al., 2000 for an example of considerable variation in cone mosaics between different species), the analysis of Keeley and Reese (2018) was not applied in the other studies. Here, we have performed a corresponding analysis of the distribution of AII amacrine cells at multiple eccentricities of rat retina. We confirm that also for this species, the distribution of AII amacrine cells appears to be regular, when analyzed by conventional spatial statistics, but, in fact, cannot be distinguished from random distributions matched by density and constrained by soma size.

## Materials and methods

### Animals and general aspects

The use of animals in this study was carried out under the approval of and in accordance with the regulations of the Animal Laboratory Facility at the Faculty of Medicine at the University of Bergen (accredited by AAALAC International). Albino rats (Wistar HanTac, bred in-house) had *ad libitum* access to food and water and were kept on a 12/12 light/dark cycle. Two wholemounts from two animals were used for immunolabeling and quantitative analysis. One wholemount was from a 7-week old female rat and one wholemount was from a 4-week old male rat, henceforth referred to as Retina-1 and Retina-2, respectively. The animals were deeply anaesthetized with isoflurane (IsoFlo vet 100%; Abbott Laboratories) in 100% O_2_ and killed by cervical dislocation. After removing the eyes, the retinas were dissected out (in HEPES-buffered extracellular solution) and used for immunolabeling.

### Immunocytochemical labeling of retinal wholemounts

Before removing the eye from the orbit, we used a marker (Penol) to place a spot of permanent ink (xylene free) on the dorsal part of the eye. After the eye had been enucleated and opened, a small incision was made in the dorsal part of the retina, choroid and sclera before dissecting the retina from the eye cup. To flatten the retina, we made four radial incisions from the periphery almost to the center and transferred the retina onto the nongridded surface of nitrocellulose filter paper (Millipore, cat. number HABG01300). Together with the attached retina, the filter paper was placed on a piece of folded tissue paper (Kimwipe). A few drops of HEPES-buffered extracellular solution were added from above and allowed to soak through. For fixation, a few drops of 4% paraformaldehyde in 0.1 m phosphate buffer (PB; 0.081 m Na_2_HPO_4_/0.019 m NaH_2_PO_4_, pH 7.4) were added from above and allowed to soak through. This procedure was repeated two to three times, after which the filter paper with the attached retina was transferred to a larger volume of 4% paraformaldehyde in 0.1 m PB and fixed for 30 min at room temperature. The retina was then washed six times (10 min each) in 0.01 m phosphate-buffered saline (PBS; 0.01 m PB with 8.76 g NaCl and 0.2 g KCl per liter, pH 7.4) and incubated overnight at 4°C in antibody incubation solution consisting of PBS with 5% normal goat serum (Sigma-Aldrich), 0.5% Triton X-100 (Sigma-Aldrich), and 0.05% NaN_3_. The retina was then incubated for four nights (at 4°C) with primary antibody (guinea pig anti-parvalbumin, diluted 1:1000) in antibody incubation solution (with 0.2% Triton X-100). Both retinas were also immunolabeled with an antibody against ankyrin-G, but this was reported in Liu et al. (2021) and will not be further commented on here. After incubation, the retina was washed six times (10 min each) in PBS and incubated overnight (at 4°C) with secondary antibodies diluted 1:1000 in antibody incubation solution (with 0.2% Triton X-100). Secondary antibodies were purchased from Thermo Fisher Scientific and for the immunolabeling for parvalbumin, we used goat anti-guinea pig coupled to Alexa 488 (#A11073). After this incubation, the retina was washed six times (10 min each) in PBS and mounted in Vectashield (refractive index 1.45; Vector Laboratories, cat. number H-1000) between a microscope slide and a precision coverslip (0.17 mm thickness; Karl Hecht Assistent, cat. number 1014/5024) separated by spacers made of small pieces of coverslip glass (~0.17 mm thickness) glued to strips of 0.12 mm thick imaging spacer disks (“SecureSeal,” Electron Microscopy Sciences, cat. number 70327-13S).

### Antibody characterization

The primary antibody against parvalbumin was a polyclonal antibody raised in guinea pig against amino acids 1–133 (Synaptic Systems, cat # 195004; RRID AB_2156476). The antibody has been validated by the supplier, as indicated in the supplier’s data sheet which showed a band of the respective molecular weight of the protein detected, as revealed by Western blot.

### Confocal microscopy and image acquisition

For imaging the immunolabeled wholemount preparations, we used a TCS SP8 confocal microscope (Leica) equipped with a ×63 glycerol immersion objective (HC PL APO Glyc corr CS2, 1.3 NA; Leica) and HyD detectors. The image stacks were acquired at 8‑bit resolution and the acquisition was controlled by LAS X software (Leica). We used the “white light” laser and acousto-optic filters to obtain an excitation laser line of 488 nm (for Alexa 488) and the emission bandwidth was set to 500–550 nm. The laser intensity for the acquisition channel was adjusted to maximize the dynamic range, with minimal saturation at the highest intensities. We used the Navigator module of the Leica LAS X software to acquire a total of 16 image stacks arranged roughly along two orthogonally oriented lines across the retina (see section “Results”). Each high-resolution image stack was acquired as a series of optical slices (260–548 slices in each stack, each slice 5352 × 5352 pixels; 246.03 × 246.03 *μ*m^2^) by sequential scanning (between lines) of the different channels. For each frame (corresponding to an acquisition channel), each line was scanned two times and accumulated to increase the signal-to-noise ratio (SNR). The confocal pinhole was set to 0.5 Airy units (calculated for 580 nm light). To obtain well-sampled image stacks that could be processed with deconvolution (see section “Image deconvolution”), images were sampled at a rate slightly higher than the ideal Nyquist rate. The Nyquist sampling distance in the lateral direction was calculated as
(1)

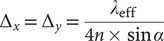

and for the axial direction, the Nyquist sampling distance was calculated as
(2)

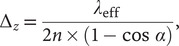

where 
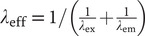
 , *λ*_ex_ is the wavelength of the excitation light, *λ*_em_ is the wavelength of the emission light, *n* is the lens medium refractive index (1.45 for the glycerol used for immersion), and *α* is the half-aperture angle of the objective (reviewed by Heintzmann, 2006; see also https://svi.nl/NyquistRate). The XY pixel size was ~46 nm and the focal plane interval was ~150 nm, sufficient to satisfy Nyquist rate sampling according to the stated equations.

In addition to the high-resolution image stacks, we also used the Navigator module of the Leica LAS X software to acquire a number of low-resolution images that tiled the entire retina (with 10% horizontal and vertical overlap between tiles). Each low-resolution image was acquired at a single focal plane (512 × 512 pixels; 246.03 × 246.03 *μ*m^2^).

### Image deconvolution

To remove noise (effectively increasing the SNR) and decrease axial and lateral blurring, we digitally deconvolved each image stack with Huygens (version 18 and 19, 64-bit, Scientific Volume Imaging; RRID:SCR_014237). Huygens reassigned out-of-focus light with a theoretically calculated point spread function, using the classic maximum likelihood estimation (CMLE) deconvolution algorithm. For each image stack, we estimated an optimal value for the user-selectable SNR parameter by repeating the deconvolution for several values of the SNR while keeping all other parameters and settings constant (see Zandt et al., 2017). For additional details, see Liu et al. (2021).

### Morphological reconstruction and analysis

We performed quantitative morphological segmentation of fluorescently labeled cell bodies in the immunolabeled wholemounts with the help of computer-aided neuronal tracing software (Neurolucida 360, version 2018 and 2019, 64-bit, MBF Bioscience; RRID:SCR_016788; Glaser & Glaser, 1990), running on a PC equipped with a graphics tablet (Cintiq 22HD, Wacom). Segmentation was performed independently by J.H.L. and D.O.P. and by a team consisting of M.K.H., M.S.F.G., and Y.G.

To segment the cell bodies of AII amacrine cells, we first selected the image slice with the largest cross-sectional area (for each cell) and manually traced the contour of the cell body. In addition to AII amacrine cells, antibodies against parvalbumin also label a population of non-AII, widefield amacrine cells in this location of the rat retina. To distinguish between AII amacrines and the widefield amacrines, we used three morphological criteria (Wässle et al., 1993). First, the intensity of labeling is lower for AII amacrines than for the widefield amacrines. Second, the cell bodies of the widefield amacrines are located slightly more distally in the retina (i.e., closer to the outer plexiform layer) compared to AII amacrines. Third, in contrast to the AIIs which have a thick (parvalbumin-positive) apical dendrite that extends vertically into the inner plexiform layer, the cell bodies of the widefield amacrine cells display a number of (parvalbumin-positive) processes that run laterally for some distance before they descend toward stratum 5 (S5) of the inner plexiform layer (for a detailed description, see Liu et al., 2021).

After we had segmented all cell bodies of immunolabeled AII amacrine cells in an image stack, we used Neurolucida 360 to automatically position a marker within each cell body contour. For each cell, the marker was located at the XYZ coordinate of the center-of-mass (centroid) of the corresponding contour. The segmentation results were processed with built-in analysis functions of Neurolucida Explorer (versions 2018 and 2019, 64-bit, MBF Bioscience; RRID:SCR_017348). IGOR Pro (version 8 and 9, WaveMetrics; RRID:SCR_000325) was used to compute the Dirichlet (Voronoi) domains (including their areas) and the nearest-neighbor distances for the population of XY coordinates corresponding to the centroids of the AII amacrine cell bodies. To compute the nearest-neighbor distances, we only took into account the XY coordinates. The regularity index (Wässle & Riemann, 1978) for the population of AII cell bodies in a given image stack was calculated as the ratio between the mean and s.d. of the Dirichlet domain areas and the nearest-neighbor distances. We used MATLAB (version 2019a, The Mathworks; RRID:SCR_001622) to calculate the number of nearest neighbors for each cell, corresponding to the number of edges in each Dirichlet domain.

### Density estimates

For morphological segmentation of parvalbumin-labeled AII cell bodies in a given image stack, we counted all contours fully included within the boundary (frame) of the stack. Unfortunately, this will underestimate the spatial density. To compensate for this, we also counted immunolabeled cell bodies intersected by the top or left edge of the image frame, as long as they were considered to unequivocally belong to AII amacrine cells. In a few cases where we were uncertain if cell bodies belonged to AII amacrines or not, we included every other such cell in the total count of AIIs.

### Analysis of nearest-neighbor distance distributions

For the AII amacrines in a given image stack, we generated histograms of nearest-neighbor distance distributions (bin width set to 1 *μ*m) and fitted each histogram with a Gaussian function (IGOR Pro):
(3)

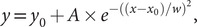

where *y*_0_ is the baseline (fixed at 0), *A* is the amplitude, and *w* (width) is equal to 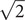 × s.d. From the number of AIIs in an image stack, we also calculated the expected probability density function for a randomly distributed population with the same spatial density (cf. Wässle & Riemann, 1978):
(4)

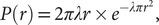

that is, the probability of finding the nearest neighbor at a distance *r* from an arbitrarily chosen point in a random point pattern with density equal to *λ.* Our data are represented in absolute and not relative frequency histograms, where the integral is equal to the total number of points in a given region. Thus, we multiplied the probability density distribution *P(r)*, where the integral is equal to 1, with the total number of points (*n*) in a given region before the corresponding function was compared with a given observed distribution:
(5)

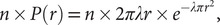

where the density λ is given by *n*/*A* and *A* is the area of each acquired region (246.03 × 246.03 *μ*m^2^).

### Computer simulations of nearest-neighbor distributions

To compare the distribution of nearest-neighbor distances observed for a given region with that for random distributions, we performed simulations (IGOR Pro). For each simulation run, we generated a random distribution of the observed number of cells within an image region (XY) of the same size as used during acquisition (246.03 × 246.03 *μ*m^2^). Each cell body was represented as a circle and the location was represented by the XY coordinates of the center of the circle. When a cell was added, its location was determined by generating two random numbers (for the X and Y center coordinates) with equal probability within the limits of the side length of a stack. The center location was also constrained by the location and size of the other cell bodies to prevent overlap. For each simulation cycle of adding a cell body, the diameter was randomly selected from a Gaussian distribution with the same mean and s.d. as observed for the segmented AII amacrine cells in a given image stack. For a segmented cell body, the apparent diameter was calculated as the diameter of a circle with the same area.

### General data analysis and presentation

For data analysis and visualization, we used Huygens, Neurolucida Explorer, IGOR Pro, MATLAB, and Excel. Density recovery profiles (DRP), based on two-dimensional point autocorrelograms, were generated (IGOR Pro) as described by Rodieck (1991). Experimental data are presented as mean ± s.d. (*n* = number of cells or points).

## Results

### Density and distribution of AII amacrine cells

**Fig. 1.**
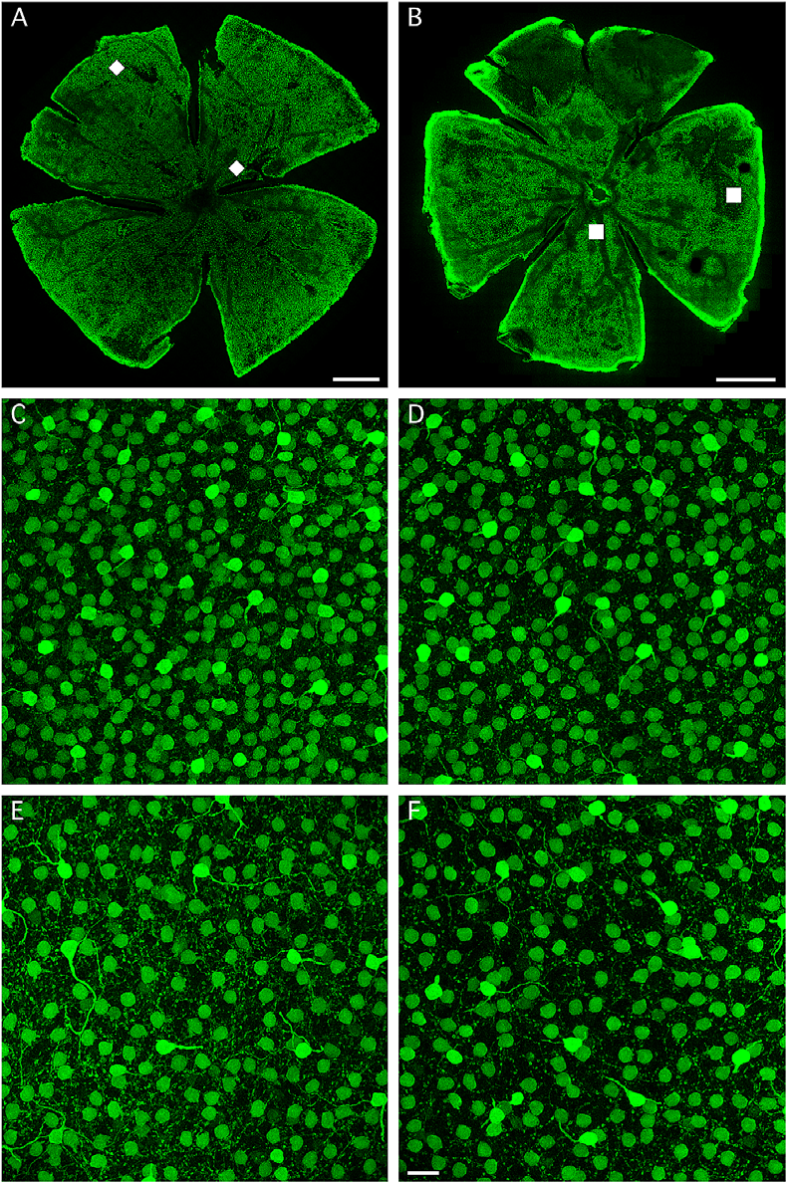
Immunolabeling of parvalbumin-containing neurons in rat retina. (**A**,**B**) Composite of low-resolution tiles (512 × 512 pixels; 246.03 × 246.03 *μ*m^2^; 944 images) of wholemount retinas immunolabeled for parvalbumin (from two animals; left “Retina-1” from 7-week old female, right “Retina-2” from 4-week old male). Each tile was acquired at a single focal plane. Because the focal plane was constant for all tiles, the labeling intensity appears uneven in different regions. The white squares correspond to the size, location and orientation (in the XY plane) of the high-resolution confocal image stacks illustrated in (**C**–**F**; **C** and **E** for Retina-1 in **A**; **D** and **F** for Retina-2 in **B**). Each wholemount retina flattened by four radial incisions that divided the retina into quadrants (different orientation for Retina-1 and Retina-2). Scale bar = 1 mm (**A**,**B**). (**C**–**F**) Maximum intensity projection (MIP) of horizontal (XY) slab of high-resolution confocal image stack (MIPs in **C** and **D** from central retina, MIPs in **E** and **F** from peripheral retina). The borders of the slab along the *Z* (depth) axis were set to encompass all parvalbumin-labeled cell bodies located proximally in the inner nuclear layer (slab thickness ~16.5 *μ*m in **C**, ~19.5 *μ*m in **D**, ~14.2 *μ*m in **E**, and ~ 19.5 *μ*m in **F**). AII amacrines have relatively weakly labeled cell bodies. The more strongly labeled cell bodies belong to a type of widefield amacrine cell, many of which display a relatively thick process that sprouts in a lateral direction. Scale bar = 20 *μ*m (**C**–**F**).

**Fig. 2.**
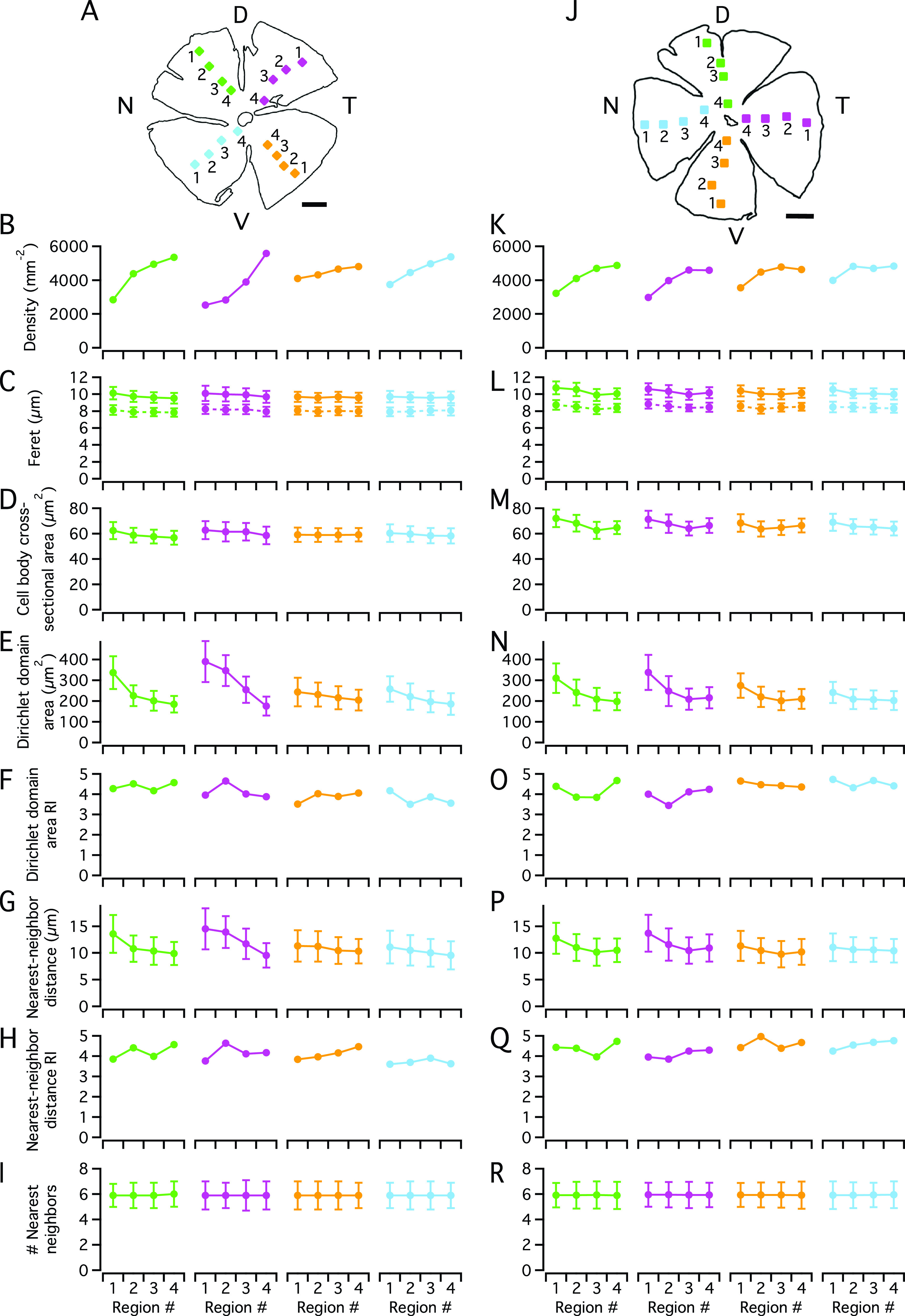
Distribution and morphological properties of AII amacrine cells in wholemount retina immunolabeled for parvalbumin. (**A**) Schematic figure of Retina-1 (same as in Fig. 1A) and the location of the 16 high-resolution confocal image stacks used for counting and morphological analysis. Here and later, the size of each colored square corresponds to the relative size of the image stack (drawn to scale, *X* × *Y* = 246.03 × 246. 03 *μ*m^2^). Each square (image stack) is numbered from 1 (most peripheral region) to 4 (most central region), and the orientation of the retina is indicated by capital letters denoting *D*orsal, *V*entral, *N*asal, and *T*emporal (**A**,**J**). Scale bars = 1 mm (**A**,**J**). (**B**) Spatial density of cell bodies as a function of retinal quadrant and eccentricity (regions 1–4), with retinal quadrant and eccentricity indicated by color and region number. (**C**) Feret maximum (continuous lines) and Feret minimum (broken lines) of the maximum cross-sectional area of the cell bodies (as seen in the XY plane of each image stack). Here and later, values are plotted as mean ± s.d. (**D**) Cross-sectional area of the cell body in the XY plane, measured in the focal plane with the maximal projection area. (**E**) Area of Dirichlet domains for the population of XY coordinates (center-of-mass locations of cell bodies) within each region. (**F**) Regularity index (RI) for the Dirichlet domain areas in (**E**), calculated as the ratio between the mean and s.d. for each image stack. (**G**) Nearest-neighbor distance for the population of XY coordinates within each region. (**H**) Regularity index for the nearest-neighbor distances in (**G**), calculated as in (**F**). (**I**) Number of nearest neighbors for the cells in each region (estimated as the number of edges for the corresponding Dirichlet domains). (**J**) As (**A)**, for Retina-2 (same as in Fig. 1B). (**K**–**R**) As (**B**–**I**), for Retina-2.

In this study, we immunolabeled two wholemount retinas (each from a separate rat, see section “Materials and methods”) for parvalbumin, a calcium-binding protein that serves as a marker for AII amacrine cells in rat retina (Wässle et al., 1993). Immunolabeling for parvalbumin also labels a population of widefield amacrine cells with cell bodies in the inner nuclear layer, but these can be (relatively) easily distinguished from AII amacrine cells based on the intensity of labeling, the relative position of the cell bodies, and the morphology of the proximal dendritic processes (see section “Materials and methods”). For any given region of the retina, the AII cells are more weakly labeled than the parvalbumin-positive widefield amacrine cells (Wässle et al., 1993). Fig. 1A and 1B shows composite low-resolution overviews of the two wholemount retinas immunolabeled for parvalbumin. From both preparations, we acquired 16 image stacks at higher resolution (see Fig. 2 below for exact locations of the stacks). High-resolution images with parvalbumin-positive cell bodies are illustrated in Fig. 1C–1F, with Fig. 1C and 1D from two centrally located image stacks (indicated by the central white squares in Fig. 1A and 1B) and Fig. 1E and 1F from two peripherally located image stacks (indicated by the peripheral white squares in Fig. 1A and 1B). Each image in Fig. 1C–1F corresponds to the maximum intensity projection (MIP) of a horizontal “slab” (substack) located close to the border between the inner nuclear and inner plexiform layers. Each image shows a large number of relatively weakly labeled cell bodies of AII amacrine cells and a smaller number of more intensely labeled cell bodies of widefield amacrine cells. It is also possible to identify several examples of parvalbumin-positive widefield amacrine cells with a number of processes emanating laterally from the cell body. Typically, one of the processes is thicker than the others and extends horizontally for some distance before descending into the inner plexiform layer. In contrast, the AII amacrines never display similar thick, parvalbumin-positive processes that sprout laterally from the cell body. The characteristic thick apical dendrites of AII amacrine cells that descend vertically into the inner plexiform layer cannot be seen in the horizontal images and slabs illustrated in Fig. 1, but were displayed in Liu et al. (2021). The smaller and even more weakly labeled structures between the labeled cell bodies in Fig. 1C–1F correspond to dendritic processes, in particular lobular dendrites and lobular appendages, of AII amacrine cells.

To analyze the distribution of AII amacrine cells in each wholemount retina, we segmented their immunolabeled cell bodies contained within each of the 16 image stacks across the retina, with four stacks in each quadrant distributed from periphery (Region #1) to center (Region #4), as illustrated in Fig. 2A and 2J. The data illustrated in Fig. 2B–2I are identical to or derived from data previously reported from our laboratory (Liu et al., 2021). From the segmented cell bodies, we calculated a series of morphological properties, including density, Feret maximum and minimum, cross-sectional area, Dirichlet domain area and regularity index, nearest-neighbor distance and regularity index, and number of nearest neighbors (Fig. 2B–2I and Fig. 2K–2R). Consistent with the findings of Wässle et al. (1993) for AII amacrine cells in the rat, we observed, for both wholemounts, that the cell body density was highest in the central and dorsal retina and decreased toward the periphery, with the most pronounced gradient found in the dorsal retina (Fig. 2B and 2K). For the two retinas, the highest density was 5187 (Fig. 2B) and 4874 (Fig. 2K) cells/mm^2^. For Retina-1, the highest density was found in the stack located most centrally in the dorsotemporal retina (Fig. 2B). For Retina-2, the highest density was found in the stack located most centrally in the dorsal retina (Fig. 2K).

Cell body size was measured by Feret maximum/minimum and cross-sectional area, and displayed a center-periphery gradient with somewhat larger cell bodies toward the periphery, as well as a tendency toward slightly larger cell bodies in the dorsal compared to the ventral retina (Fig. 2C and 2D and Fig. 2L and 2M). On average, the cell bodies in Retina-2 were larger than those in Retina-1, with cross-sectional areas (Fig. 2D and 2M) of 66.1 ± 6.6 *μ*m^2^ (*n* = 4005 cells) versus 59.2 ± 6.3 *μ*m^2^ (*n* = 3895 cells; *P* < 0.0001, Student’s two-tailed *t*-test). The eccentricity-dependence of cell density was paralleled by similar (but opposite) relationships for Dirichlet domain area and nearest-neighbor distance. Specifically, the Dirichlet domain area and the nearest-neighbor distance increased from the center toward the periphery, concomitant with the corresponding decrease in density (Fig. 2E, 2G, 2N, and 2P). Similar to the observations for cell body density, we observed the larger gradients in the dorsal compared to the ventral retina. We calculated a regularity index (mean/s.d.) for both Dirichlet domain area and nearest-neighbor distribution, with the former preferred by some authors because it takes into account a cell’s relation to all its neighbors, not only its nearest (e.g., Keeley & Reese, 2018; see also discussion in Reese, 2008*b*). For Dirichlet domain area, the average regularity index was 4.04 ± 0.35 (range 3.50–4.65; *n* = 16 image stacks) in Retina-1 (Fig. 2F) and 4.28 ± 0.35 (range 3.45–4.73; *n* = 16 image stacks) in Retina-2 (Fig. 2O). For nearest-neighbor distance, the average regularity index was 4.05 ± 0.33 (range 3.60–4.63) in Retina-1 (Fig. 2H) and 4.41 ± 0.30 (range 3.85–4.96) in Retina-2 (Fig. 2Q). The latter values are very similar to that reported by Wässle et al. (1993). Despite minor variability between the different image stacks, there was no systematic difference between central and peripheral regions for either index (Fig. 2F and 2O and Fig. 2H and 2Q). For both wholemount retinas, the average number of nearest neighbors (estimated as the number of edges in the Dirichlet domains) was remarkably constant (~5.9–6) across the entire retina (Fig. 2I and 2R).

**Fig. 3.**
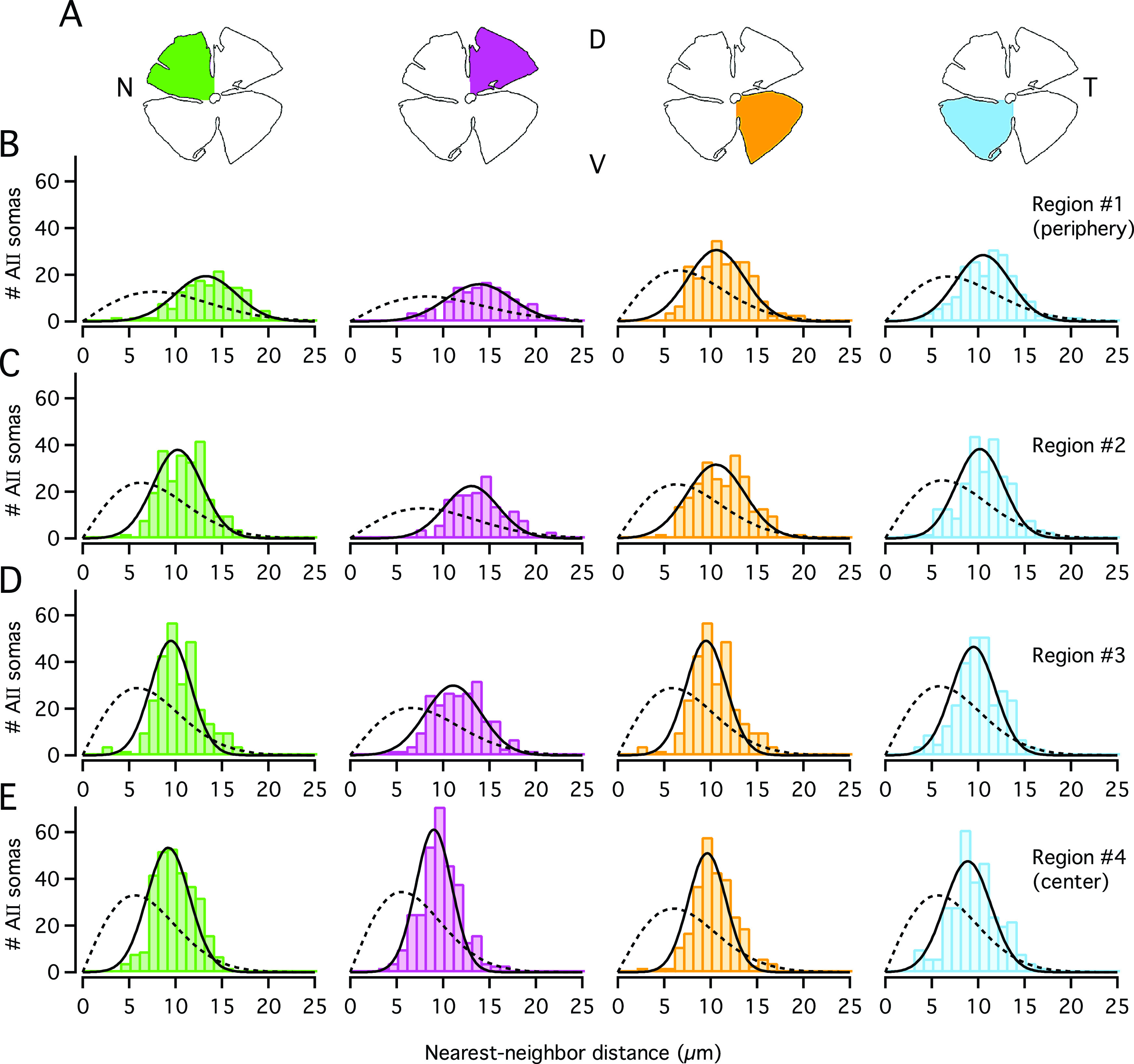
Distribution of nearest-neighbor distances for AII cell bodies in Retina-1. (**A**) Schematic figures of wholemount retina. For the graphs in (**B**–**E**), colors correspond to quadrant colors in (**A**) and region numbers correspond to those in Fig. 2A. (**B**) Frequency histograms (bin width 1 *μ*m) displaying the distribution of nearest-neighbor distances for cell bodies within the most peripheral region (#1) in each quadrant. Each histogram was fitted with a Gaussian function (eqn. (3); continuous black line). The broken black line in each panel shows the expected probability density function (multiplied by the total number of cells for a given region; eqn. (5)) for a randomly distributed population of points with the same spatial density, but where the exclusion zones imposed by cell body size are ignored (for details, see section “Materials and methods”). (**C**–**E**) As in (**B**), but for regions #2, #3, and #4 (from periphery to center) in each retinal quadrant.

**Fig. 4.**
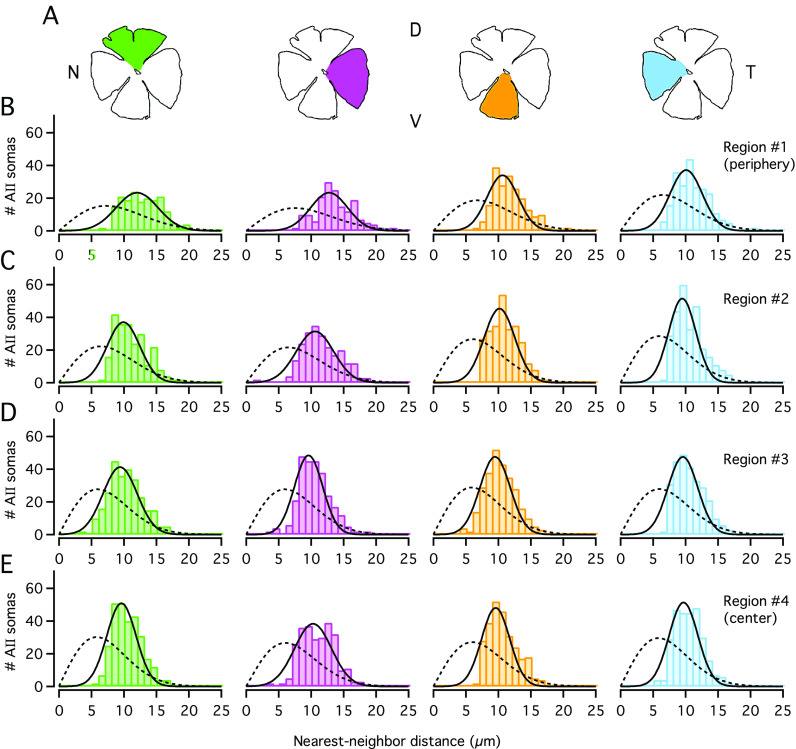
Distribution of nearest-neighbor distances for AII cell bodies in Retina-2. (**A**) Schematic figure of wholemount retina. (**B**–**E**) Frequency histograms (bin width 1 *μ*m) displaying the distribution of nearest-neighbor distances for cell bodies within the four different regions, from #1 in periphery to #4 in center (as in Fig. 3B–3E).

In addition to calculating the regularity index from the mean and s.d. of the nearest-neighbor distances, we analyzed the corresponding distributions in more detail. For each retina, we have illustrated the nearest-neighbor distance frequency distributions for all 16 image stacks distributed across the retina (Figs. 3 and 4). It is readily apparent that all the frequency distribution histograms were well fitted by Gaussian functions (Figs. 3 and 4). This was the case both for the most central (#4), the most peripheral (#1), as well as the intermediate (#2, #3) regions within each quadrant. In contrast, the theoretical functions that displayed the expected random distributions for the identical spatial densities (cf. Wässle & Riemann, 1978) did not provide adequate fits to the observed distributions (Figs. 3 and 4).

### Simulation of random distributions of AII amacrine cell bodies

The results for the nearest-neighbor distance distributions for AII cell bodies suggested that these cells are regularly (i.e., nonrandomly) distributed and that this pertains to all regions and eccentricities of the retina (Figs. 3 and 4). However, as pointed out by Reese and coworkers (e.g., Keeley & Reese, 2018; Keeley et al., 2020), these results cannot be taken as evidence for a nonrandom distribution, as they do not take into account that soma size will constrain the possible location of cell bodies and that this might impact the apparent regularity for a given soma size and density. To investigate this for the distribution of AII cell bodies in our material, we performed simulations where we generated random distributions of cell bodies with size and density similar to those observed experimentally (see section “Materials and methods” for details).

**Fig. 5.**
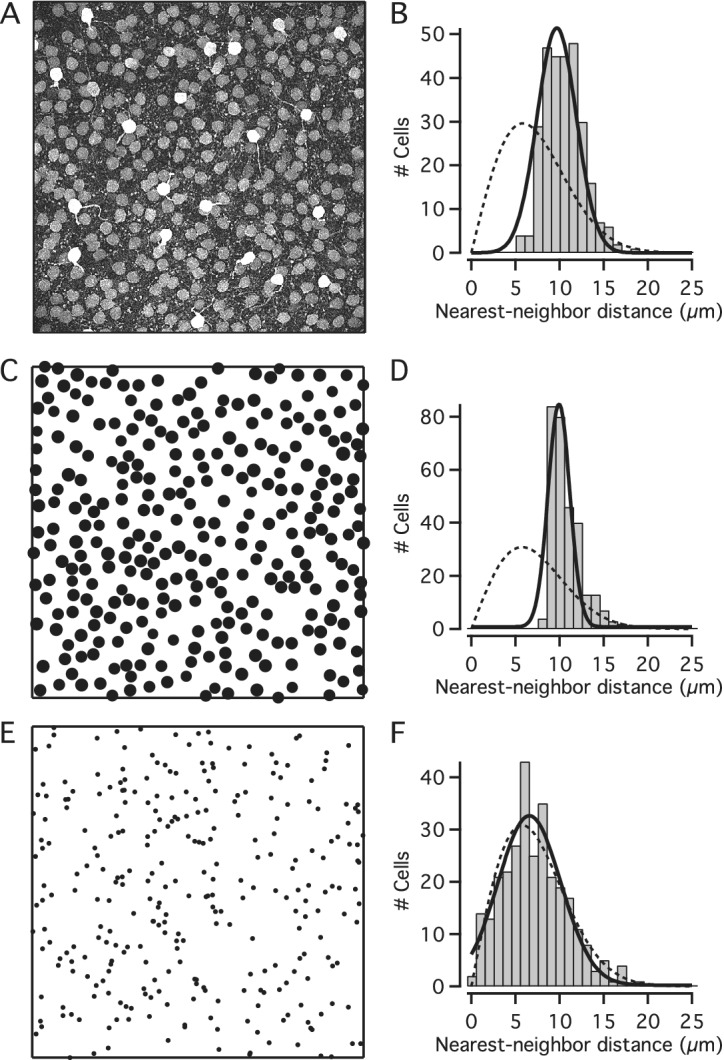
Nearest-neighbor distance distributions for real and simulated populations of cells. (**A**) MIP of horizontal (XY) slab of confocal image stack (corresponding to region #4 in nasal quadrant of Retina-2; *X* × *Y* = 246.03 × 246.03 *μ*m^2^; slab thickness ~ 27 *μ*m). Notice weakly labeled AII amacrines and strongly labeled widefield amacrines. Manual segmentation of AII cell bodies found *n* = 292 cells, average apparent cell body diameter: 9.01 ± 0.39 *μ*m. (**B**) Frequency histogram (bin width 1 *μ*m) for the distribution of nearest-neighbor distances for cell bodies in (**A**), fitted with a Gaussian function (eqn. (3); continuous black line). The broken black line shows the expected probability density function (multiplied by the total number of cells; eqn. (5)) for a randomly distributed population of points with the same spatial density, but where the exclusion zones imposed by cell body size are ignored. (**C**) Localization and size of cell bodies generated by simulating a random distribution (single trial), with density and diameter (average, s.d.) taken from the population of cells in (**A**). During the simulation, the exclusion zones imposed by cell body size were respected, such that cell bodies were allowed to touch, but not overlap. (**D**) Frequency histogram (bin width 1 *μ*m) for the distribution of nearest-neighbor distances for cell bodies in (**C**), fitted with a Gaussian function (eqn. (3); continuous black line). The broken black line shows the expected probability density function (multiplied by the total number of cells) for a randomly distributed population of points with the same spatial density, but where the exclusion zones imposed by cell body size are ignored. (**E**) Localization of cell body centers generated by simulating a random distribution (single trial), with density taken from the population of cells in (**A**). During the simulation, the exclusion zones imposed by cell body size were ignored, treating cell bodies as points. (**F**) Frequency histogram (bin width 1 *μ*m) for the distribution of nearest-neighbor distances for points in (**E**). The broken black line shows the expected probability density function (multiplied by the total number of cells) for a randomly distributed population of points with the same spatial density. The continuous black line indicates the result from fitting the histogram with a Gaussian function (eqn. (3)).

We found that simulations of random distributions of cell bodies (with diameter and spatial density as determined for a given stack) generated nearest-neighbor distance histograms that were well fitted by Gaussian functions and appeared markedly different from the expected random distributions for the identical spatial densities. The results for an individual image stack in one of the wholemounts are illustrated in Fig. 5. Fig. 5A shows a MIP of a substack (as in Fig. 1C–1F) immunolabeled for parvalbumin, with numerous AII cell bodies displaying moderate intensity of labeling and a few widefield amacrine cell bodies displaying strong intensity of labeling. The total number of AIIs in this stack was 292 and the average apparent diameter was 9.01 ± 0.39 *μ*m. The corresponding histogram of nearest-neighbor distances was well fitted by a Gaussian function and appeared distinctly different from the expected random distribution (Fig. 5B; same as Fig. 4E, Region #4). Using parameters obtained from this image stack, we performed simulations of random distributions with the same spatial density and constrained by soma size. The results from a single simulation run are illustrated in Fig. 5C and 5D. Fig. 5C shows the random distribution of cell body locations and diameters and Fig. 5D shows the nearest-neighbor distance histogram, Gaussian curve fit and expected random distribution. One property of the histograms generated by simulations consistently differed from the histograms observed for the real AII distributions, corresponding to the lack of nearest-neighbor distances less than ~7–8 *μ*m in the simulations. There are two likely explanations for this difference. First, because the real AII cell bodies tend not to be perfectly circular (see difference between Feret maximum and minimum in Fig. 2C and 2L), some nearest-neighbor distances can become quite small. Second, there is a small difference in the depth of location of real AII cell bodies in the inner nuclear and inner plexiform layers such that when projected onto the XY plane (e.g., Fig. 5A) some neighboring cell bodies display an apparent overlap, thereby giving rise to quite small nearest-neighbor distances in the XY plane. Irrespective of this, the frequency distribution of nearest-neighbor distances for the randomly generated population of (circular) cell bodies was clearly different from the expected random distribution (Fig. 5D). Apart from random variation from trial to trial, similar results were seen for every simulation run (data not shown).

We next performed identical simulations where we set the average cell body size to zero (equivalent to considering the cell bodies as points), with markedly different results. The results for a single simulation run are illustrated in Fig. 5E and 5F. Fig. 5E shows the random distribution of point (“cell body”) locations and Fig. 5F shows the nearest-neighbor distance histogram and expected random distribution. For completeness, Fig. 5F also shows the Gaussian curve fit. Importantly, in this case the frequency histogram of nearest-neighbor distances is very similar to the expected random distribution. Taken together, these results suggested that the distribution of real AII cell bodies across the retina cannot be distinguished from a random distribution when cell body size is taken into account (cf. Keeley & Reese, 2018).

### How robust is the conclusion that AII distributions cannot be distinguished from a random distribution?

The results presented in Fig. 5 strongly suggested that when a random distribution is generated without taking cell body size into account, the distribution of nearest-neighbor distances corresponds very well to the expected random distribution and is very different from the distributions observed for real AII cell bodies. The results, however, do not provide any obvious answers with respect to how much smaller the diameters and densities would have to be, compared to those observed for real AII cell bodies, before the simulation results would generate distributions similar to the expected random distributions for points. For example, the question arises if there is any possibility of observing a random nearest-neighbor distribution, if a region of the retina had lower AII cell body density or size than observed in our analysis?

**Fig. 6.**
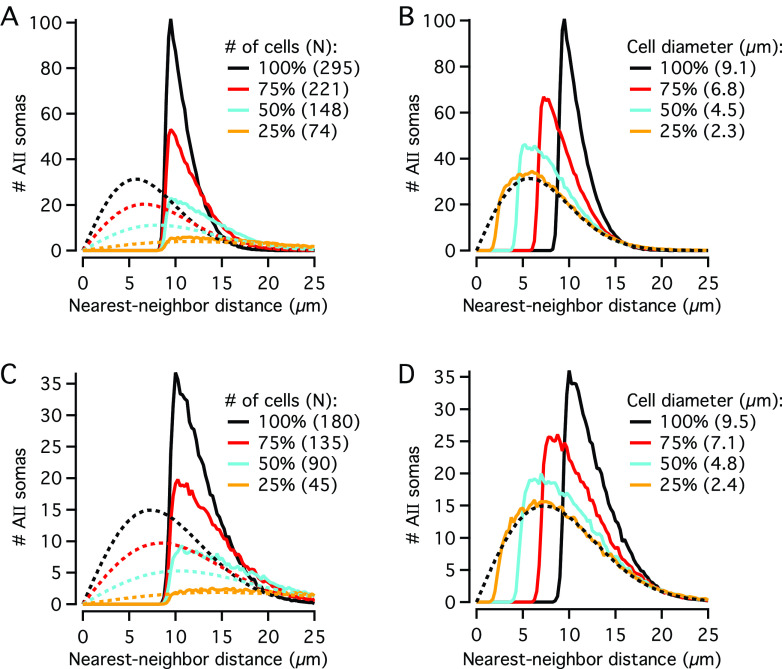
Influence of reduced density and cell body size on nearest-neighbor distance histograms for simulated random distributions. (**A**) Continuous lines display frequency distributions (bin width 0.25 *μ*m) of nearest-neighbor distances generated by simulating random distributions of cell bodies with density and diameter (average, s.d.) taken from AII amacrines in an image stack (corresponding to region #4 in dorsal quadrant of Retina‑2; *n* = 295 cells; apparent diameter = 9.07 ± 0.36 *μ*m). As indicated by the panel legend, the different colors correspond to simulations where the cell density was 100, 75, 50, and 25% of that in the image stack. Here and in (**B**–**D**), each frequency distribution is the average of 500 simulation trials, multiplied by the inverse of the bin width for direct comparison with the expected probability density functions (for a randomly distributed population of points with the same spatial density, but where the exclusion zones imposed by cell body size are ignored), as shown by the broken lines (same color code). (**B**) Continuous lines display frequency distributions (bin width 0.25 *μ*m) of nearest-neighbor distances generated by simulating random distributions of cell bodies with density and diameter (average, s.d.) taken from AII amacrines in an image stack (same as in **A**). As indicated by the panel legend, the different colors correspond to simulations where the average cell body diameter was 100, 75, 50, and 25% of that in the image stack. The broken black line shows the expected probability density function (multiplied by the total number of cells) for a randomly distributed population of points with the same spatial density. (**C**) As in (**A**), but with density and diameter (average, s.d.) taken from AII amacrines in a different image stack (corresponding to region #1 in temporal quadrant of Retina-2; *n* = 180 cells; apparent diameter = 9.52 ± 0.45 *μ*m). (**D**) As in (**B**), but for same retina as in (**C**).

To explore this question, we simulated distributions where we reduced either the cell density or the (apparent) cell diameter to 75, 50, or 25% of the values observed for a given stack. For a given simulation condition, we repeated the simulation 500 times and calculated the average frequency distribution. To generate histograms with higher resolution, we set the bin width to 0.25 *μ*m and multiplied the average histograms by 4 to compare with the expected random distributions. Fig. 6A and 6B shows the results for simulations based on an image stack corresponding to Region #4 in the dorsal quadrant of the retina displayed in Fig. 1B. For this stack, the number of AIIs was high (295 cells), but the average apparent diameter was relatively low (9.07 ± 0.36 *μ*m). It was readily apparent, however, that for both density and diameter, the values had to be reduced to much lower levels than observed for the real AIIs for the frequency histograms to match the corresponding expected random distributions. In both cases, the 50% reductions were markedly different, whereas the 25% reductions were very similar (Fig. 6A and 6B).

We also performed simulations based on a second image stack from the same retina, corresponding to Region #1 in the temporal quadrant. For this stack, the number of AIIs was lower (180 cells) and the average apparent diameter was relatively high (9.52 ± 0.45 *μ*m). Also in this case, however, it was readily apparent that the values both for density and diameter had to be markedly reduced relative to the values for the real AIIs before the distributions became similar to the expected random distribution (Fig. 6C and 6D). These results suggested that within realistic limits for cell body density and size, there is essentially no likelihood that observed distributions of AIIs in rat retina, even when genuinely regular (i.e., nonrandom), can be clearly differentiated from a random distribution.

## Discussion

The putative spatial regularity among retinal neurons of a given type has been considered important for two main reasons. First, for neurons that play a direct role in processing and analyzing the visual image, a regular distribution has in general been thought to be significant because it enables a relatively uniform contribution from each type of neuron across the retina (Reese, 2008*b*). Even when the density of a type of neuron varies between different regions (dorsal, ventral, nasal, and temporal) or when it depends on eccentricity, the size of the dendritic fields changes in the opposite manner to maintain a relatively constant coverage factor. Second, assuming that such regularity is indeed present, and that the degree of regularity is pronounced, the question arises how the regularity is established during development (Reese, 2008*a*; Reese & Keeley, 2015).

A number of studies have attempted to apply criteria to decide whether different distributions, that is, different mosaics, of a specific type of retinal neuron are regular or random. In addition, if a distribution is regular, that is, nonrandom, how regular is it? The simplest spatial statistic employed is the distribution of nearest-neighbor distances together with the corresponding regularity index (calculated as the mean divided by the s.d., i.e., the inverse of the coefficient of variation). A given distribution is then compared with the expected probability density function for a random (Poisson) distribution of points of the same density. This kind of analysis has been performed for many different types of neurons in several different species. When the distribution of AII amacrine cells was analyzed in retina of cat, rabbit, rat, macaque, mouse, and bat, it was in every case concluded that the observed mosaic displayed relatively high regularity, with a regularity index of approximately 4–5.5 (Vaney, 1985; Mills & Massey, 1991; Vaney et al., 1991; Wässle et al., 1993, 1995; Casini et al., 1995; Jeon et al., 2007; Pérez de Sevilla Müller et al., 2017).

### Morphological labeling of AII amacrine cells in different mammalian retinas

The first study that investigated the spatial distribution of AII amacrines was performed in cat retina (Vaney, 1985). In this study, the spatial distribution of AIIs was visualized by incubating retina *in vitro* with DAPI. Although DAPI labels multiple types of neurons, AIIs were identified by their location and intensity of labeling (Vaney, 1985). For rat retina, the first encompassing study was that of Wässle et al. (1993), with AIIs immunolabeled for parvalbumin. Although a small proportion of parvalbumin-labeled cells (<1%) might be misclassified as either AII or widefield amacrines, we consider it extremely unlikely that such errors will be systematic and have any noticeable impact on the overall density estimates of AIIs in rat retina. In principle, similar problems are associated with estimates of AII densities based on immunolabeling in other mammalian retinas. For example, in mouse retina immunolabeling for the transcription factor Prox1 labels more than one type of retinal cell, but only AIIs among amacrine cells (in the mature retina) which can be identified because of their specific localization within the retina (Pérez de Sevilla Müller et al., 2017).

### Distribution of AII amacrine cells across the rat retina

In both retinas that we examined, the density of AII amacrines displays a similar global pattern across the retina. The density is highest in the dorsotemporal retina and the corresponding quadrants also display the largest gradient with respect to the difference between central and peripheral retina. For the two retinas examined, the ratio between the highest (central) and lowest (peripheral) density within a given quadrant was 1.5 and almost 3, respectively. For the ventronasal retina, there was much less of a center-periphery gradient. The center-periphery gradient of the dorsotemporal retina is much more pronounced than what was observed for mouse retina by Pérez de Sevilla Müller et al. (2017). Although it is difficult to compare the various density estimates directly because of methodological differences, the overall densities seem fairly similar between rat and mouse retina, with ~4000 cells/mm^2^. This result is also similar to the density measurements reported by Keeley and Reese (2018) for a number of retinas from different mouse strains. They analyzed a total of 13 retinas, but only one field for each retinal quadrant.

The peak density observed for the two retinas in our study (~5200 and ~4900 cells/mm^2^) is somewhat lower than the peak densities reported for the two retinas analyzed by Wässle et al. (1993; 7000 and 7500 cells/mm^2^, respectively). As suggested in a previous study from our laboratory (Liu et al., 2021), the difference could be due to the exact locations sampled, the extent of shrinkage, and age differences. Importantly, whereas the spatial density varies as a function of retinal region and eccentricity, with reduced density toward the periphery, the number of nearest neighbors stays essentially constant across the retina. The results presented by Wässle et al. (1993) fall within the range of cell densities and soma sizes explored in the simulations presented in our Fig. 6. For example, the distribution analyzed in Fig. 6 of Wässle et al. (1993) displayed a density of ~2400 cells/mm^2^. The soma size was not reported, but from their figure the average diameter can be estimated as ~8.7 *μ*m. Accordingly, simulations of random soma distributions with density and size matched to the data of Wässle et al. (1993) would be indistinguishable from their experimentally observed distributions. Thus, we conclude that there is no fundamental difference between our results and those of Wässle et al. (1993).

### Soma distribution of AII amacrines: Regular or random?

For the two retinas analyzed in our study, we calculated a regularity index for the distribution of AIIs in each field, both for the nearest-neighbor distance distribution and for the Dirichlet domain area distribution. It has been argued that because the nearest-neighbor distance distribution only takes into account the relation between an individual neuron and one of its neighbors, the Dirichlet domain area is a more general measure which takes into account the relation to all the neighbors (reviewed by Reese, 2008*b*). For our material, however, the results for both regularity indices were very similar, with most values in the range of 4–5 and with little variation between different regions, different eccentricities, and retinas.

In addition to nearest-neighbor distance distribution, other, more complicated methods have been developed to analyze whether a mosaic displays regular or random distribution. The method known as the DRP (Rodieck, 1991) is based on a two-dimensional point autocorrelogram. In contrast to nearest-neighbor analysis, it is better suited for addressing whether a mosaic displays higher-order (“lattice-like”) periodicity, because it takes into account the distance from any given cell to all other cells in the mosaic (reviewed by Reese & Keeley, 2015). In common with most post-receptoral mosaics, the AII amacrine mosaic in mouse retina does not display any evidence for such periodicity (Keeley & Reese, 2018). When we analyzed rat AII mosaics (real and simulated) with the DRP method, the only deviation from a random distribution was an exclusion zone at the origin of the autocorrelogram (data not shown), in agreement with Keeley and Reese (2018). For random distributions constrained by soma size, it is obvious that this will only affect the distributions close to the origin of the autocorrelogram. For in-depth discussions of these methods of mosaic analysis, see reviews by Reese and Keeley (2015) and Keeley et al. (2020).

The most important question, however, is whether our results for the distribution of AII amacrines in rat retina differ in any substantial way from the corresponding results for AIIs in mouse retina (Keeley & Reese, 2018), where it was concluded that the distribution could not be discriminated from a random distribution with the same density and constrained by soma size. When we compared the distributions obtained for AIIs in specific regions with simulated distributions matched by density and constrained by soma size, with average and standard deviation drawn from the real population of AIIs for the same region, we reached the same conclusion as Keeley and Reese (2018). In every case examined, it was not possible to discriminate between the real and simulated random distributions. In contrast, if the simulations were performed with point elements, that is, cells with no spatial extent, the simulated random distributions appeared identical to those obtained for the expected probability density functions for random distributions (Poisson). Because the constraint imposed by the physical size of the real somas will have an increasing influence when the spatial density increases (as less space becomes available for additional cells), we also explored the robustness of our conclusion by performing additional simulations where we reduced either the density or the average soma size. In both cases, the distributions only approached the probability density distributions expected for a random distribution of points when density and soma size were reduced to approximately 25% of the real values. This was observed both for a central region with high spatial density and a peripheral region with lower spatial density.

The fact that the real distributions of AII amacrine cells cannot be discriminated from simulated random distributions matched for density and constrained by soma size does not by itself mean that the distribution is inherently random. Arguably, the important point is how the final distribution is generated during development of the retina. With the constraint that the AII cell bodies are essentially confined to a single tier of the inner nuclear layer, the real distributions could potentially be generated by a series of mechanisms without an intrinsic ability to establish regularity. For the relatively high density of the population of AII amacrine cells, there is no clear need for a mechanism to explicitly establish regularity in the distribution. Future work will be required to decipher the actual mechanisms involved during development (cf. Reese & Keeley, 2015).
